# Assessing the combined effect of surface topography and substrate rigidity in human bone marrow stem cell cultures

**DOI:** 10.1002/elsc.202200029

**Published:** 2022-09-13

**Authors:** Sofia Ribeiro, Eugenia Pugliese, Stefanie H. Korntner, Emanuel M. Fernandes, Manuela E. Gomes, Rui L. Reis, Alan O'Riordan, Yves Bayon, Dimitrios I. Zeugolis

**Affiliations:** ^1^ Medtronic Sofradim Production Trevoux France; ^2^ Regenerative Modular & Developmental Engineering Laboratory (REMODEL) and Science Foundation Ireland (SFI) Centre for Research in Medical Devices (CÚRAM) National University of Ireland Galway (NUI Galway) Galway Ireland; ^3^ 3B's Research Group I3Bs – Research Institute on Biomaterials Biodegradables and Biomimetics University of Minho Headquarters of the European Institute of Excellence on Tissue Engineering and Regenerative Medicine AvePark Parque de Ciência e Tecnologia Zona Industrial da Gandra Barco Guimarães Portugal; ^4^ ICVS/3B's – PT Government Associate Laboratory Braga/Guimarães Portugal; ^5^ Tyndall National Institute Cork Ireland; ^6^ Regenerative Modular & Developmental Engineering Laboratory (REMODEL) Charles Institute of Dermatology Conway Institute of Biomolecular & Biomedical Research and School of Mechanical & Materials Engineering University College Dublin (UCD) Dublin Ireland

**Keywords:** biodegradable polyesters, stem cell differentiation, substrate stiffness, surface topography

## Abstract

The combined effect of surface topography and substrate rigidity in stem cell cultures is still under‐investigated, especially when biodegradable polymers are used. Herein, we assessed human bone marrow stem cell response on aliphatic polyester substrates as a function of anisotropic grooved topography and rigidity (7 and 12 kPa). Planar tissue culture plastic (TCP, 3 GPa) and aliphatic polyester substrates were used as controls. Cell morphology analysis revealed that grooved substrates caused nuclei orientation/alignment in the direction of the grooves. After 21 days in osteogenic and chondrogenic media, the 3 GPa TCP and the grooved 12 kPa substrate induced significantly higher calcium deposition and alkaline phosphatase (ALP) activity and glycosaminoglycan (GAG) deposition, respectively, than the other groups. After 14 days in tenogenic media, the 3 GPa TCP upregulated four and downregulated four genes; the planar 7 kPa substrate upregulated seven genes and downregulated one gene; and the grooved 12 kPa substrate upregulated seven genes and downregulated one gene. After 21 days in adipogenic media, the softest (7 kPa) substrates induced significantly higher oil droplet deposition than the other substrates and the grooved substrate induced significantly higher droplet deposition than the planar. Our data pave the way for more rational design of bioinspired constructs.

**Abbreviations**
AFM: atomic force microscopy
ALP: alkaline phosphatase
BM: basal media
BSA: bovine serum albumin
CD: cluster of differentiation
DMEM: Dulbecco's Modified Eagle Medium
DSC: differential scanning colorimetry
EDTA: ethylenediaminetetraacetic acid
FACS: fluorescence‐activated cell sorting
FBS: fetal bovine serum
FOV: fields of view
GAG: glycosaminoglycan
hBMSCs: human bone marrow stem cells
HBSS: Hanks’ balanced salt solution
NIL: nanoimprint lithography
P/S: penicillin streptomycin
PBS: phosphate buffered saline
PFA: paraformaldehyde
PGCL 10/90: poly(glycolide‐*co*‐ε‐caprolactone)
PLTMC 80/20: poly(lactide‐*co*‐(trimethylene carbonate)
RIN: RNA integrity
RMS: root mean square
SEM: scanning electron microscope
SF: serum free
TCP: tissue culture plastic
αMEM: α‐minimal essential medium

## 1 INTRODUCTION

In vivo, cell fate is determined by biochemical and biological signals provided by soluble factors [1, 2] and biophysical signals provided by the surrounding extracellular matrix (ECM) [3, 4]. In vitro, although soluble factors have shown promise in controlling cell fate [5, 6], the literally infinite number of potential permutations on biochemical and/or biological media supplements, concentrations, combinations, and timings has restricted their use. To this end, the use of biophysical cues has been advocated [7, 8, 9] and, due to simplicity, surface topography [10, 11] and substrate stiffness [12, 13] have been the subject of very many investigations in the quest to either maintain permanently differentiated cell phenotype (e.g., tenocyte [14, 15], chondrocytes [16, 17], osteoblasts [18, 19]) or to direct stem cells towards a specific lineage (e.g., tenogenic [20, 21], chondrogenic [22, 23], osteogenic [24, 25], adipogenic [26, 27]). Considering though that cells in vivo are subjected simultaneously to multiple signals, mono‐factorial approaches are unlikely to yield a functional output (e.g., 85/15 poly(lactic‐*co*‐glycolic acid) with anisotropic grooves, although resulted in upregulation of tendon genes, due to its high rigidity, also induced upregulation of bone and cartilage genes [28]), which has triggered investigations into multi‐factorial tissue engineering [29, 30, 31].

The combined effect of surface topography and substrate stiffness in cell function has been the subject of many investigations, the findings of which have been recently summarized [32, 33]. It is worth noting though that most research has been conducted with non‐degradable polymers (such as polydimethylsiloxane, PDMS, [34, 35, 36, 37]), which are of little value in the development of regenerative medicine implantable devices. Considering that implantable device/target tissue and local cells mechanical properties mismatch is still associated with implant failure [38, 39, 40, 41], it is imperative to assess the combined effect to substrate rigidity and surface topography on biodegradable devices using relevant human cell populations. Interestingly, only one study has assessed the influence of surface topography (i.e., nano‐grating of 150 nm groove depth, 250 nm groove width, and 250 nm distance between grooves and nano‐holes of 225 nm diameter, 400 nm pitch, and 300 nm depth) and substrate rigidity (i.e., 62, 128, and 204 MPa) on biodegradable polymers [i.e., poly(ε‐caprolactone), poly(glycolic acid), poly(lactic acid)] using human bone marrow stem cells (hBMSCs) [42].

Herein, we employed nanoimprint lithography (NIL) to create aliphatic polyester [poly(glycolide‐*co*‐ε‐caprolactone) (PGCL 10/90) with elastic modulus of 7 kPa and poly(lactide‐*co*‐(trimethylene carbonate) (PLTMC 80/20) with elastic modulus of 12 kPa] substrates with planar and anisotropic topography (groove width: 1.08 ± 0.09 μm and 1.01 ± 0.03 μm; groove depth: 1.46 ± 0.12 μm and 1.43 ± 0.10 μm; distance between groves: 1.64 ± 0.08 μm and 1.78 ± 0.05 μm, respectively). Planar tissue culture plastic (TCP) (∼3 GPa elastic modulus) and planar and grooved polymeric substrates were used to study hBMSC morphology, basic cellular function, and differentiation towards osteogenic, chondrogenic, tenogenic, and adipogenic lineages.

## 2 MATERIALS AND METHODS

### 2.1 Materials

The aliphatic polyesters used were PGCL 10/90 composed of 9.6% glycolide and 90.4% caprolactone (PGCL 10/90) with elastic modulus of 7 ± 3 kPa and PLTMC 80/20 composed of 79.1% lactide and 20.9% trimethylene carbonate monomer (PLTMC 80/20) with elastic modulus of 12 ± 3 kPa. The elastic modulus values were determined in previous study of the group using atomic force microscopy (AFM) [43]. The polymers were produced by Medtronic (North Haven, USA). All tissue culture plastics were purchased from Sarstedt (Ireland). All chemicals, cell culture media, and reagents were purchased from Sigma–Aldrich (Ireland), unless otherwise stated.

### 2.2 Fabrication of polymeric substrates

The polymeric substrates were obtained by compression molding as described before [43]. Briefly, a thermal presser (Carver 3856 CE, Carver, USA) was heated close to the polymer melting temperature (PGCL 10/90: 90°C, PLTMC 80/20: 220°C). The polymer pellets were placed between two metal sheets covered by Teflon sheets and subjected to a minimum pressure of 1 bar for 5 min. Subsequently, the system was gradually cooled down (10°C/min) to approximately 30°C. The fabrication method was performed under controlled temperature and humidity conditions. Settings were selected to obtain polymeric substrates of 200 μm in thickness. The produced substrates were stored in sealed aluminum bags in desiccants at 4°C until use.

The topography was induced on the polymeric substrates in a cleanroom laboratory using established protocols [18, 28]. Briefly, silicon master stamp with grooved topography (groove width of 0.78 ± 0.04 μm; groove depth of 1.46 ± 0.05 μm; distance between the grooves of 2.31 ± 0.04 μm) were fabricated via a photolithography process, followed by reactive ion etching (RIE). Silicon wafers (3.0 × 3.0 cm^2^) were spin‐coated with a positive photoresist (S1813 PR, Shipley) and then exposed using OAI Mask Aligner (Model MBA800). Following photoresist development, the master stamp was etched by RIE (Oxford ICP etcher) using CHF3 + SF6 ionized gas. The molds were salinized with 5 mM octadecyltrichlorosilane solution to enable imprint release. A NIL system (Obducat Eitre 3, Sweden) was used to produced patterned polymeric substrates, by placing a silicon mold of inverse pattern in direct contact with the polymeric substrates at an imprinting temperature of 42°C for PGCL 10/90 and 165°C for PLTMC 80/20, at a pressure of 6 bar for a period of 15 min, enabling an accurate pattern transfer. Substrates were demolded after the system temperature was cooled to 25°C.

**PRACTICAL APPLICATION**

This manuscript assessed human bone marrow stem cell response on aliphatic polyester substrates as a function of grooved topography and rigidity. Low rigidity (alone or combined with anisotropic topography) substrates favor tenogenic and adipogenic induction of human bone marrow stem cells (hBMSCs). On the other hand, high rigidity (alone or combined with anisotropic topography) substrates favor osteogenic and chondrogenic induction of hBMSCs. Such substrates hold great potential for the medical device and cell culture technologies sectors.

### 2.3 Morphology analysis of polymeric substrates

The morphology of all the produced substrates was characterized using a high‐resolution field emission scanning electron microscope (SEM) with focused ion beam (Auriga Compact, Zeiss, Germany). AFM (AFM Dimension Icon, Bruker, USA) was also used in PeakForce Tapping (ScanAsyst) mode in air. AFM cantilevers (ScanAsyst‐Air, Bruker), made of silicon nitride, were used with a spring constant of 0.4 N/m and frequency of 70 kHz. The images, with a scan size of 20 × 20 μm, were analyzed using a commercial AFM software (Bruker) and the surface roughness was measured as the root mean square (RMS) roughness. RMS was calculated using the Z‐sensor height signal. A total of 18 locations (six locations of three replicates) were analyzed per formulation.

### 2.4 Thermal properties analysis of polymeric substrates

Differential scanning colorimetry (DSC) analysis was conducted using a DSC 1 Star System (Mettler Toledo, USA), which was programmed to perform two heating cycles, with a cooling intermediated step. The following temperatures heating protocols were used for each polymer: PGCL 10/90: ‐75°C to 75°C and PLTMC 80/20: 30°C to 190°C. All tests were performed at a heating rate of 10°C/min. The mass of the analyzed sample was between 5 and 6 mg. The second heating cycle was used to determine the glass transition temperature (*T*_g_), enthalpy of cold crystallization (Δ*H*_cc_), melting temperature (*T*_m_), enthalpy of melting (Δ*H*_m_), and crystallinity content (*X*_c_), which was calculated using the following formula: The crystallinity *X*_c_ of the polyesters was determined by the following formula: *X*_c_ = [(Δ*H*_m_–Δ*H*_cc_)/(Δ*H*_mc_w)] × 100, where Δ*H*_m_ is the melting enthalpy (J/g) of the sample; Δ*H*_cc_ is the cold crystallization enthalpy (J/g); Δ*H*_mc_ is the melting enthalpy of the 100% crystalline poly(lactide) (93.7 J/g) [44] and poly(ε‐caprolactone) (136.1 J/g) [45]; and w is the mass fraction of predominant monomer in the composite.

### 2.5 Contact angle analysis of polymeric substrates

Static contact angle measurements were obtained using the sessile drop method and an OCA 15 Plus goniometer (DataPhysics Instruments, Germany) with a high‐performance image processing system (DataPhysics Instruments, Germany). Three microliters of either deionized water or diiodomethane were added using a motor driven syringe at room temperature (RT). The values of surface free energy were calculated by the Owens, Wendt, Rabel, and Kaelble (OWRK) method [46] that discerns polar and dispersive components of the surface energy, using the SCA20 version 2 software (DataPhysics Instruments, Germany). At least six measurements of each condition were performed per group.

### 2.6 Cell isolation and culture

hBMSCs were isolated according to standard protocols [47]. Briefly, bone marrow, obtained from the iliac crest, was purchased from Caltag (UK). The bone marrow was washed in phosphate buffered saline (PBS) and subsequently plated on TCP in α‐minimal essential medium (αMEM) supplemented with 10% fetal bovine serum (FBS) and 1 % penicillin streptomycin (P/S). Cells were cultured at 37°C in a humidified atmosphere of 5% CO_2_. After 7 days in culture, the non‐adherent cells were removed by several washes with PBS and the adherent cells (passage 0) were cultured at 80% to 90% confluency. For passaging, cells were detached using trypsin‐ethylenediaminetetraacetic acid (EDTA). For tenogenic differentiation only, cells at passage 3 were starved in medium without serum for one full passage (hBMSCs‐SF). Prior to cell seeding, the polymeric substrates were sterilized with ethylene oxide at Medtronic (USA). hBMSCs or hBMSCs‐SF at passage 4 were detached using trypsin‐EDTA, washed with PBS, and centrifuged at 800 g for 5 min. The cell pellet was resuspended in αMEM supplemented with 10% FBS and 1% P/S. Subsequently, 100 μl of the cell suspension were poured on top of the substrates, which were placed at the bottom of 24 well plates. The cells were allowed to attach for 2.5 h prior to adding 900 μl of complete basal medium. The media were changed every other day. Cells seeded on TCP served as control group.

### 2.7 Flow cytometry analysis

Cells were incubated with various combinations of fluorochrome‐labelled antibodies (Table S1) to assess cluster of differentiation (CD) mesenchymal stem cell markers (CD31, CD44, CD45, CD73, CD90, CD105, CD146, along with their respective isotype controls) according to the manufacturer's instructions (BD Stemflow™, UK). In brief, cells were washed with cold PBS, trypsinized for 5 min and αMEM with 10% FBS was added to neutralize trypsin's activity. Cells were collected, washed with 2% FBS in PBS, centrifuged at 800 g for 5 min and the supernatant was removed. Cells were resuspended in 2% FBS in PBS and strained through a 40 μm cell strainer. Cells were counted and diluted to a concentration of 1 million cells per ml in 2% FBS in PBS. Subsequently, ∼100,000 cells were placed in each tube and stained with the appropriate volume of fluorochrome‐labelled antibodies for 30 min at RT. Cells were washed twice in PBS and resuspended in 2% FBS in PBS. Sytox blue (Invitrogen, Ireland) was used as viability dye. Cells were analyzed using a fluorescence‐activated cell sorting (FACS) equipment (BS FACSCanto™ II Cell Analyser, BD Biosciences, UK) and the percentage of positive cell populations were calculated using FlowJo software v10 (TreeStar Inc., USA). Flow cytometry analysis revealed that hBMSCs (Figure S1) and hBMSCs‐SF (Figure S2) expressed high percentage of positive and low percentage of negative mesenchymal stem cell markers.

### 2.8 Cell morphometry and cell proliferation analysis

For assessing in vitro cell morphology, cells were seeded onto polymeric substrates, at a density of 500 cells/cm^2^ and were cultured in αMEM supplemented with 10% FBS and 1% P/S. After 3 and 7 days of culture, cells were fixed with 4% paraformaldehyde (PFA) for 2 h at 4°C, blocked with 3% bovine serum albumin (BSA) in PBS for 30 min at RT, and permeabilized with 0.2% Triton X‐100 for 5 min at RT. The samples were incubated with rhodamine labeled phalloidin (66 μM in PBS, 1:200, Invitrogen, Ireland) for 2 h at RT to stain cytoskeleton and with Hoechst 33342 solution (20 mM in PBS, 1:5000, Invitrogen, Ireland) to stain nuclei for 5 min at RT. Fluorescent images were captured using an Olympus IX‐81 inverted fluorescence microscope (Olympus Corporation, Japan) at 10x magnification and analyzed with ImageJ (NIH, USA). 3 replicates of each group were imaged and 5 fields of view (FOV) were taken from each replicate (a total of 15 images were analyzed per experimental group). The following cell shape characteristics were measured for each fitted ellipse: aspect ratio (major axis/minor axis) and Feret's diameter (the longest distance between any two points along the selection boundary). Aspect ratio was used to evaluate nuclear elongation with a higher aspect ratio indicating increased elongation. Nuclear orientation/alignment was determined by the angle (0‐180 degrees) of the Feret's diameter. Cell proliferation analysis was performed by nuclei counting normalized to the area of the image.

### 2.9 Cell metabolic activity analysis

Metabolic activity was assessed in using the alamarBlue™ assay (Invitrogen, USA) according to the manufacturer's protocol. In brief, after 14 and 21 days of culture, cells were washed with Hanks’ balanced salt solution (HBSS) and alamarBlue™ solution (10% alamarBlue™ in HBSS) was added. After 4 h of incubation at 37°C, absorbance was measured in triplicate at excitation wavelength of 550 nm and emission wavelength of 595 nm using Varioskan Flash spectral scanning multimode reader (ThermoFisher Scientific, UK). Cell metabolic activity was normalized to cell number. Nuclei were counted to obtain cell number.

### 2.10 Osteogenic differentiation analysis

Cells were seeded on the various substrates at density of 20,000 cells/cm^2^. Cells were allowed to attach and spread for 48 h in αMEM supplemented with 10% FBS and 1% P/S (basal media [BM]). Osteogenesis was induced with 10 mM β‐glycerophosphate disodium salt hydrate, 100 nM dexamethasone, 50 μM ascorbic acid‐2‐phosphate in αMEM supplemented with 10% FBS and 1% P/S. The differentiation media were changed every 3 days up to 21 days of incubation.

Osteogenic differentiation was assessed by quantification of calcium deposition using the StanBio Calcium Liquicolour™ Kit (ThermoFisher Scientific, Ireland) after 14 and 21 days of culture in basal and osteogenic media. Samples were digested with 0.5 M HCl overnight at 4°C. A standard curve was generated using 0, 1, 5, 10, 30, 50 and 100 mg/ml calcium concentrations in 0.5 M HCl. Ten microliters of cell lysate or standard and 200 μl of working solution were added to 96 well plate. The working solution was composed of 1 to 1 color reagent to base reagent. Absorbance at 550 nm was measured using a Varioskan Flash spectral scanning multimode reader (ThermoFisher Scientific, UK) and the amount of calcium per well was calculated using calcium standards and normalized to the amount of DNA, which was quantified using Quant‐iT™ PicoGreen dSDNA assay kit (Invitrogen, Ireland) according to the manufacturer's protocol. Briefly, after 14 and 21 days of culture, DNA was extracted using 3 freeze‐thaw cycles after adding 250 μl of nucleic acid‐free water per well. One hundred microliters of sample were transferred into a 96‐well plate. A standard curve was generated using 0, 5, 10, 25, 50, 100, 500 and 1,000 ng/ml DNA concentrations. One hundred microliters in ultra‐pure water of a 1:200 dilution of Quant‐iT™ PicoGreen reagent were added to each sample and the plate was read using a micro‐plate reader (Varioskan Flash, ThermoFisher Scientific, UK) with excitation wavelength of 480 nm and emission wavelength of 525 nm.

Alkaline phosphatase (ALP) activity was assessed after 14 and 21 days of culture in basal and osteogenic media by lysing the cells with deionized water, frozen them at ‐80°C, and thawing them at RT (the freeze‐thaw was done twice). Twenty microliters of the cell lysate and standard were incubated with 80 μl of 1‐Step™ p‐nitrophenyl phosphate Substrate Solution (ThermoFisher Scientific, UK). After 30 min of incubation at 37°C, the reaction was stopped by adding 100 μl of 0.05 M NaOH. Absorbance was measured at 405 nm using a Varioskan Flash spectral scanning multimode reader (ThermoFisher Scientific, UK). The amount of p‐nitrophenol was calculated using p‐nitrophenol (10 mM 4‐nitrophenol) standards and the units of the enzyme were calculated by dividing the μmoles of p‐nitrophenol produced by time (min) and then by normalizing them to the amount of DNA.

### 2.11 Chondrogenic differentiation analysis

Cells were seeded on the various substrates at density of 20,000 cells/cm^2^. Cells were allowed to attach and spread for 48 h in αMEM supplemented with 10% FBS and 1% P/S (BM). Chondrogenesis was induced with 100 nM dexamethasone, 100X ITS+1(insulin, transferrin, sodium selenite, linoleic‐BSA) liquid media supplement, 40 μg/ml L‐proline, 50 μg/ml ascorbic acid‐2‐phosphate, 10 ng/ml TGF‐β3 in high glucose Dulbecco's Modified Eagle Medium (DMEM) supplemented with 1% P/S. The differentiation media were changed every 3 days up to 21 days of incubation.

Chondrogenic differentiation was analyzed by quantifying sulphated glycosaminoglycan (GAG) deposition using the 1,9‐dimethylmethylene blue (DMMB) method (Blyscan Sulphated Glycosaminoglycan Assay, Biocolor, UK) after 14 and 21 days of culture in basal and chondrogenic media. Briefly, samples were digested in a solution of 50 μg/ml proteinase K in 100 mM K_2_HPO_4_(pH 8.0) overnight at 56°C. Subsequently, proteinase K was inactivated by heating the sample for 10 min at 90°C [48]. The cell lysates were centrifuged and the supernatants were collected. A standard curve was generated using bovine tracheal chondroitin 4‐sulfate standard. Fifty microliters of cell lysate were transferred to a clean Eppendorf tube and 1 ml of dye reagent was added. After agitation, the samples were centrifuged and the supernatants were discarded without disrupting the pellet. Five hundred microliters of dye dissociator were added to the samples and mixed. Absorbance was measured at 656 nm using a Varioskan Flash spectral scanning multimode reader (ThermoFisher Scientific, UK). The GAG content of the pellets was normalized to the amount of DNA.

### 2.12 Tenogenic differentiation analysis

After one full passage in serum free (SF) media, hBMSCs‐SF maintained in Mesencult‐ACF Plus Medium (Stem Cells Technologies, UK) and incubated at 37°C in a 5% CO_2_ and 5% O_2_ environment. Cells were seeded on the various substrates at density of 20,000 cells/cm^2^. Cells were allowed to attach and spread for 48 h in Mesencult‐ACF Plus Medium. Tenogenic differentiation was induced with Mesencult tenogenic differentiation media (Stem Cells Technologies, UK). The differentiation media were changed every 3 days up to 21 days of incubation.

Tenogenic differentiation was assessed by gene expression of early growth response 1(EGR1), early growth response 2(EGR2), scleraxis (SCX), collagen type I (COL1A1), collagen type III (COL3A1), mohawk homeobox (MKX), tenascin c (TNC) and tenomodulin (TNMD) markers by qPCR after 3 and 14 days of culture. Total RNA was isolated using the RNeasy Plus Micro Kit (Qiagen, Germany) according to the manufacturer's protocol. Briefly, samples were disrupted in Buffer RLT and homogenized. Ethanol was then added to the lysate and the samples were transferred to the RNeasy Micro spin column. All bind, wash and elution steps were performed by centrifugation in a microcentrifuge. Total RNA was retained in the membrane (bind step), contaminants were efficiently washed away (wash step) and high‐quality RNA was eluted in RNase‐free water (elution step). RNA concentration and purity were determined using a NanoDrop 1,000 (ThermoFisher Scientific, Ireland). Samples with RNA purity values of 260/280 ratio ∼1.8 and 260/230 ratio ∼1.9 were used for qPCR experiments. RNA integrity (RIN) was assessed with an Agilent 2100 Bioanalyser (Agilent Technologies, Ireland). Samples with RIN values of > 8 were used for qPCR experiments. Samples with RIN < 8 were excluded from the study. 1 μg total RNA was reverse transcribed using the iScript™ cDNA Synthesis Kit (Bio‐Rad Laboratories, Ireland). 5 ng cDNA were subsequently analyzed by qPCR on a StepOnePlus™ Real‐Time PCR System (Thermo Fisher Scientific, Ireland), using TaqMan primer probe assays (IDT, Belgium) and TaqMan Gene Expression Mastermix (ThermoFisher Scientific, Ireland). The TaqMan primer probe assays used are listed in Table S2. The amplification conditions were 50°C for 2 min, 95°C for 10 min, followed by 40 cycles of 95°C for 15 sec and 60°C for 1 min. qBasePlus v. 2.4(Biogazelle NVBelgium) was used to perform geNorm analysis to determine the optimal number of reference genes. CQ values were analyzed and normalized relative quantities (NRQs) were calculated by normalizing the data to the expression of three validated endogenous control genes (EIF2B1, HPRT1, TBP) with qBasePlus v. 2.4 (Biogazelle NVBelgium) [49].

### 2.13 Adipogenic differentiation analysis

Cells were seeded on the various substrates at density of 20,000 cells/cm^2^. Cells were allowed to attach and spread for 48 h in αMEM supplemented with 10% FBS and 1% P/S (BM). Adipogenesis was induced with 1 μM dexamethasone, 1 μM rosiglitazone, 0.5 mM 3‐isobutyl‐1‐methyl‐xanthine and 10 μg/ml insulin in high glucose DMEM supplemented with 10% FBS and 1% P/S. After 3 days of adipogenic induction, media were switched to adipogenic maintenance media (10 μg/ml insulin in high glucose DMEM supplemented with 10% FBS and 1% P/S). The differentiation media were changed every 3 days up to 21 days of incubation.

Adipogenic differentiation was evaluated by Oil Red O staining. After 14 and 21 days of culture, the cells were fixed with 4% PFA for 20 min at 4°C. A 0.5% Oil red O stock solution was dissolved in denoised water and added to the samples for 20 min at RT. The samples were washed 3 times in PBS. For quantification of Oil Red O staining, the dye was extracted pipetting 100% isopropanol over the surface of the wells. Then, the solution was centrifuged at 500 g for 2 min to remove debris and the absorbance was measured at 520 nm using a Varioskan Flash plate reader (ThermoFisher Scientific). Results were normalized to DNA quantity.

### 2.14 Statistical analysis

Data are expressed as mean ± standard deviation. All experiments were conducted at least in three independent replicates. Statistical analysis was performed using GraphPad v6.01(GraphPad Software Inc., USA). One‐ or two‐way ANOVA was used for multiple comparisons and a Tukey post hoc test was used for pairwise comparisons after confirming that the samples followed a normal distribution (Kolmogorov‐Smirnov test) and had equal variances (Bartlett's and Levene's test for homogeneity of variances). When either or both of these assumptions were violated, nonparametric tests were used for multiple (Kruskal–Wallis test) and pairwise (Mann–Whitney test) comparisons. Statistical significance was accepted at *p* < 0.05.

## 3 RESULTS

### 3.1 Surface topography and physicochemical analyses of polymeric substrates

**FIGURE 1:**
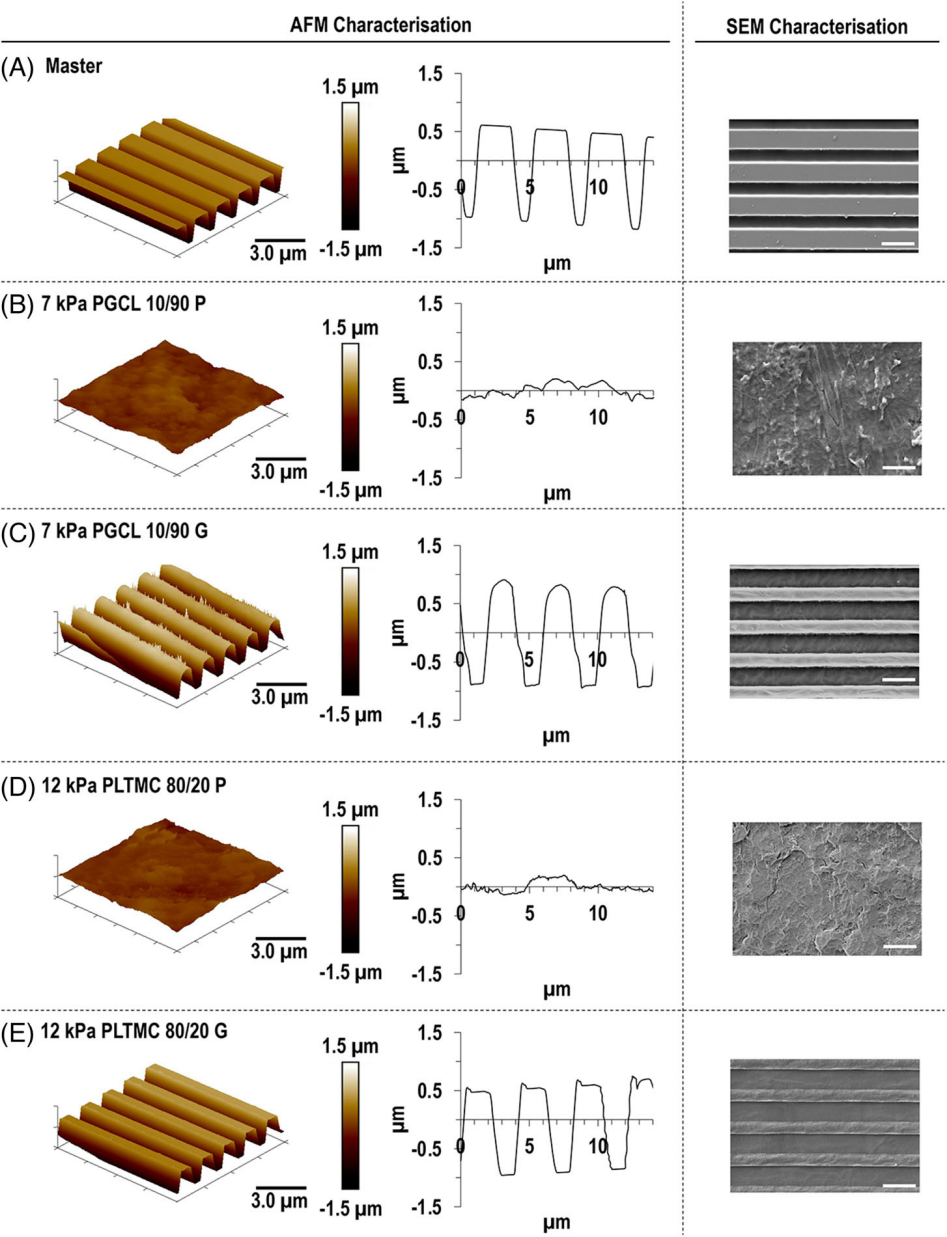
AFM imaging and lines profile and SEM imaging of silicon master (A), PGCL 10/90 P (B), PLTMC 80/20 P (C), PGCL 10/90 G (D), and PLTMC 80/20 G (E) substrates show that NIL process successfully transferred the grooved topography onto the polymeric substrates. AFM scale bar 3.0 μm. SEM scale bar 4 μm.

Qualitative SEM and AFM analyses of the grooved and planar substrates revealed a clear anisotropic and isotropic, respectively, surface topography (Figure 1). Quantitative surface analysis demonstrated that the planar/isotropic PGCL 10/90 and PLTMC 80/20(PGCL 10/90 P and PLTMC 80/20 P) substrates had RMS of 89.98 ± 51.55 nm and 75.76 ± 48.43 nm, respectively. The grooved/anisotropic PGCL 10/90 and PLTMC 80/20(PGCL 10/90 G and PLTMC 80/20 G) substrates had groove width of 1.08 ± 0.09 μm and 1.01 ± 0.03 μm, groove depth of 1.46 ± 0.12 μm and 1.43 ± 0.10 μm and distance between groves of 1.64 ± 0.08 μm and 1.78 ± 0.05 μm, respectively, revealing a good fidelity to the dimensionality of the master, which had groove width of 0.78 ± 0.04 μm, groove depth of 1.46 ± 0.05 μm and distance between the grooves of 2.31 ± 0.04 μm.

DSC analysis (Figure S3 and Table 1) revealed no apparent thermal differences (*p >* 0.05) within a polymer as a function of planar and grooved surface topography, apart from the *T*_m_ that was significantly (*p* < 0.05) increased following imprinting. The planar and grooved PGCL 10/90 substrates exhibited significantly (*p <* 0.05) lower *T*_g_, Δ*H*_cc_, and *T*_m_ and significantly (*p <* 0.05) higher *X*_c_ than their respective planar and grooved PLTMC 80/20 substrates. No significant (*p >* 0.05) differences were observed in Δ*H*_m_ between the two polymers, independently of the surface structure.

**TABLE 1:** Thermal properties of the second heating curve demonstrates that *T*_m_ that was significantly (*p* < 0.05) increased following imprinting

		**PGCL 10/90 P**	**PGCL 10/90 G**	**PLTMC 80/20 P**	**PLTMC 80/20 G**
Thermal properties	*T*_g_ (°C)	‐52 ± 1	‐50 ± 1	51 ± 0	51 ± 0
	Δ*H*_cc_ (J/g)	ND	ND	30 ± 2	32 ± 5
	*T*_m_ (°C)	31 ± 1	46 ± 1	166 ± 0	174 ± 1
	Δ*H*_m_ (J/g)	46 ± 2	44 ± 3	40 ± 1	41 ± 5
	*X*_c_	10 ± 1	10 ± 0	3 ± 1	3 ± 1
Contact angle (°)	Water	86 ± 5	96 ± 2	77 ± 2	95 ± 4
	Diiodomethane	37 ± 5	59 ± 5	48 ± 3	56 ± 1
Surface energy (mN/m)	Surface energy	39 ± 0	28 ± 0	37 ± 0	29 ± 0
	Dispersive component	37 ± 0	27 ± 0	29 ± 0	27 ± 0
	Polar component	3 ± 0	2 ± 0	8 ± 0	2 ± 0

*Note*: PGCL 10/90 P and PGCL 10/90 G substrates exhibited the lowest (*p <* 0.05) *T*_g_, Δ*H*_cc_ and *T*_m_ values and the highest (*p <* 0.05) *X*_c_ value. No significant (*p >* 0.05) differences were observed in Δ*H*_m_. *N* = 4. Contact angles and surface energy of the polymeric substrates produced in this study. *N* = 4.

Abbreviations: *T*_g_, glass transition temperature; Δ*H*_cc_, enthalpy of cold crystallization; *T*_m_, melting temperature; Δ*H*_m_, enthalpy of melting; *X*_c_, crystallinity content; ND, not detected.

Further physicochemical analysis (Table 1) of the PGCL 10/90 and PLTMC 80/20 substrates revealed that after imprinting, the contact angles in water and diiodomethane were significantly (*p <* 0.05) increased; the surface energy and the dispersive component were significantly (*p <* 0.05) decreased; and the polar component was not affected (*p >* 0.05). The planar PGCL 10/90 substrates exhibited significantly (*p <* 0.05) lower contact angles in water and diiodomethane and significantly (*p <* 0.05) higher surface energy and dispersive component than the planar PLTMC 80/20 substrates. No significant (*p >* 0.05) differences were observed between the planar PGCL 10/90 and PLTMC 80/20 substrates in the polar component.

### 3.2 Cell morphology analysis

Qualitative immunocytochemistry (Figure S4) analysis revealed that at both timepoints, the cells’ cytoskeleton adopted a random orientation on all planar substrates and a bidirectional morphology, parallel to the orientation of the grooves, on all grooved substrates. Quantitative nuclear orientation/alignment analysis (Figure S5A) revealed that cells seeded on planar substrates exhibited random orientation/alignment ‐ Whereas cells seeded on the grooved PLTMC 80/20 and PGCL 10/90 substrates exhibited ∼56% and ∼30% respectively nuclear orientation/alignment parallel to the substrate topography. Nuclear aspect ratio analysis (Figure S5B) revealed no significant (*p* > 0.05) differences between the groups.

### 3.3 Cell proliferation and metabolic activity analyses

No significant (*p >* 0.05) differences in cell proliferation (Figure S6A) and metabolic activity (Figure S6B) were observed between the groups.

### 3.4 Osteogenic differentiation analysis

**FIGURE 2:**
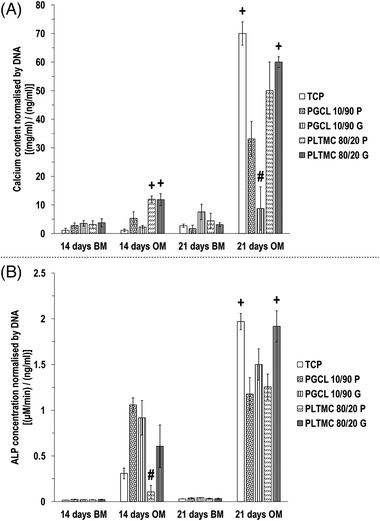
In osteogenic media on day 21, the PLTMC 80/20 G substrate exhibited significantly (*p <* 0.05) enhanced calcium deposition (A) and ALP deposition (B). + indicates significantly (*p <* 0.05) higher value than the other groups at a given timepoint. # indicates the lowest (*p <* 0.05) value at a given timepoint. *N* = 4.

In basal media (BM) on day 14 and day 21 calcium deposition (Figure 2A) and ALP concentration (Figure 2B) was very low in all groups with no biologically relevant differences between the groups. In osteogenic media on day 14, the PLTMC 80/20 substrates induced significantly (*p <* 0.05) higher calcium deposition than the other groups, and on day 21, the TCP, followed by the PLTMC 80/20 G, induced significantly (*p <* 0.05) higher and the PGCL 10/90 G induced the lowest (*p <* 0.05) calcium deposition among all groups (Figure 2A). In osteogenic media on day 14, the PLTMC 80/20 P induced the lowest (*p* < 0.05) ALP concentration and on day 21, the TCP and the PLTMC 80/20 G induced significantly (*p <* 0.05) higher ALP concentration than the other groups (Figure 2B).

### 3.5 Chondrogenic differentiation analysis

**FIGURE 3:**
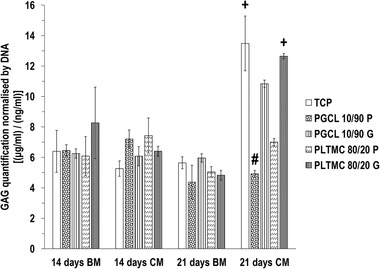
In chondrogenic media, TCP and PLTMC 80/20 G substrate induced the highest (*p <* 0.05) GAG content. + indicates significantly (*p <* 0.05) higher value than the other groups at a given timepoint. # indicates the lowest (*p <* 0.05) value at a given timepoint. *N* = 4.

In BM, on day 14 and day 21, all groups induced similar (*p >* 0.05) GAG amounts (Figure 3). In chondrogenic media on day 14, all groups induced similar (*p >* 0.05) GAG amounts and on day 21, the TCP and the PLTMC 80/20 G induced higher (*p* < 0.05), and the PGCL 10/90 P induced the lowest (*p* < 0.05) GAG amounts among all groups (Figure 3).

### 3.6 Tenogenic differentiation analysis

**FIGURE 4:**
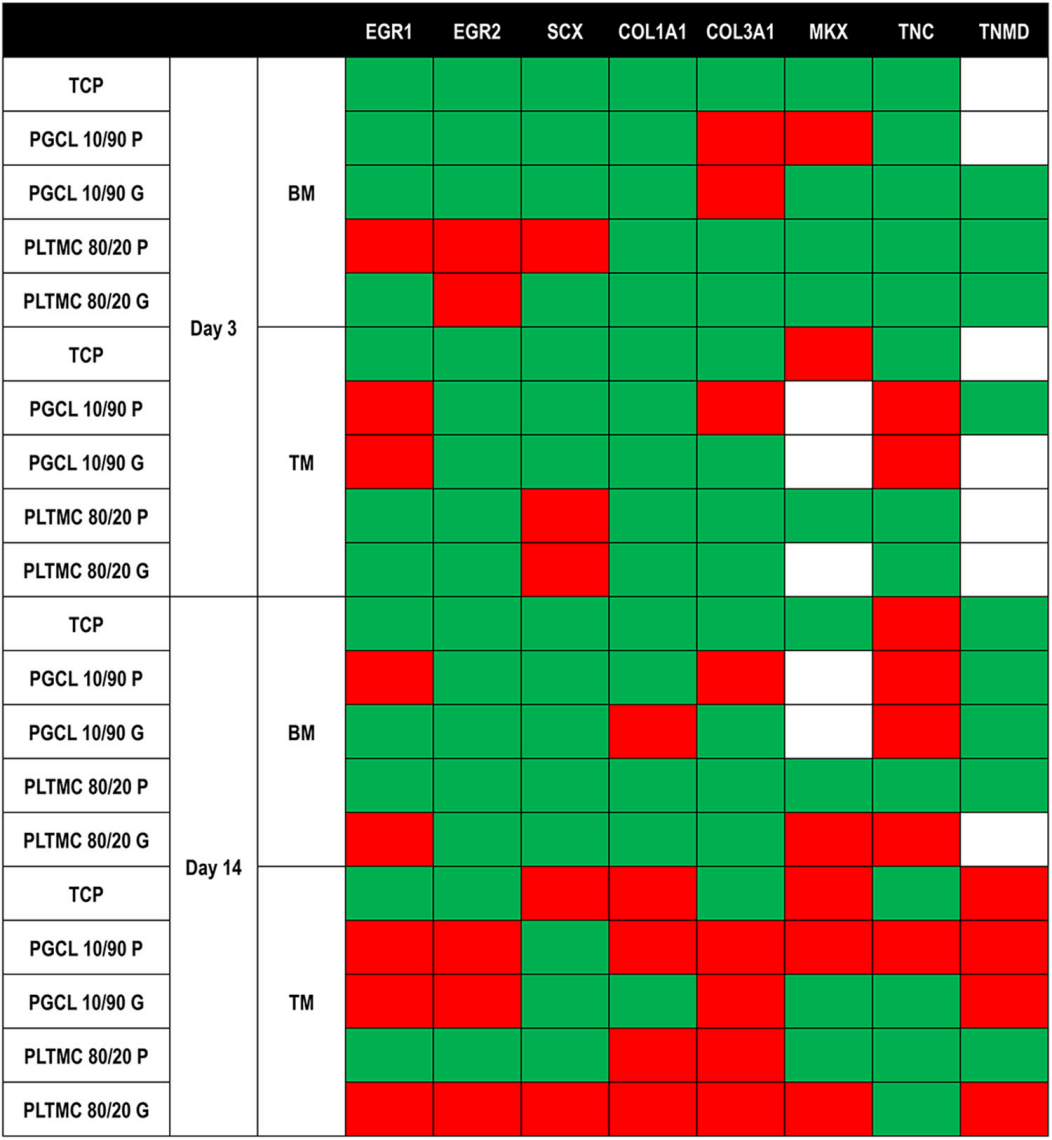
Gene analysis demonstrates that PGCL 10/90 P and PLTMC 80/20 G caused an upregulation (fold change ≥ 2.0) in the expression of tenogenic markers after 14 days of culture. Green background: Downregulated 2‐fold. Red background: Upregulated 2‐fold. White: Not detected. *N* = 4.

Gene expression analysis (Figure 4) revealed that in BM, on day 3, only the planar PLTMC 80/20 upregulated (fold change ≥ 2.0) three tenogenic genes (EGR1, EGR2, SCX) and on day 14, the planar PGCL 10/90(EGR1, COL3A1, TNC) and the grooved PLTMC 80/20(EGR1, MKX, TNC) upregulated (fold change ≥ 2.0) three tenogenic genes each. In tenogenic media, on day 3, only the planar PGCL 10/90 upregulated (fold change ≥ 2.0) three tenogenic genes (EGR1, COL3A1, SCX) and on day 14, the planar PGCL 10/90(EGR1, EGR2, COL1A1, COL3A1, MKX, TNC, TNMD) and the grooved PLTMC 80/20(EGR1, EGR2, SCX, COL1A1, COL3A1, MKX, TNMD) upregulated (fold change ≥ 2.0) seven tenogenic genes each (Figure 4).

### 3.7 Adipogenic differentiation analysis

**FIGURE 5:**
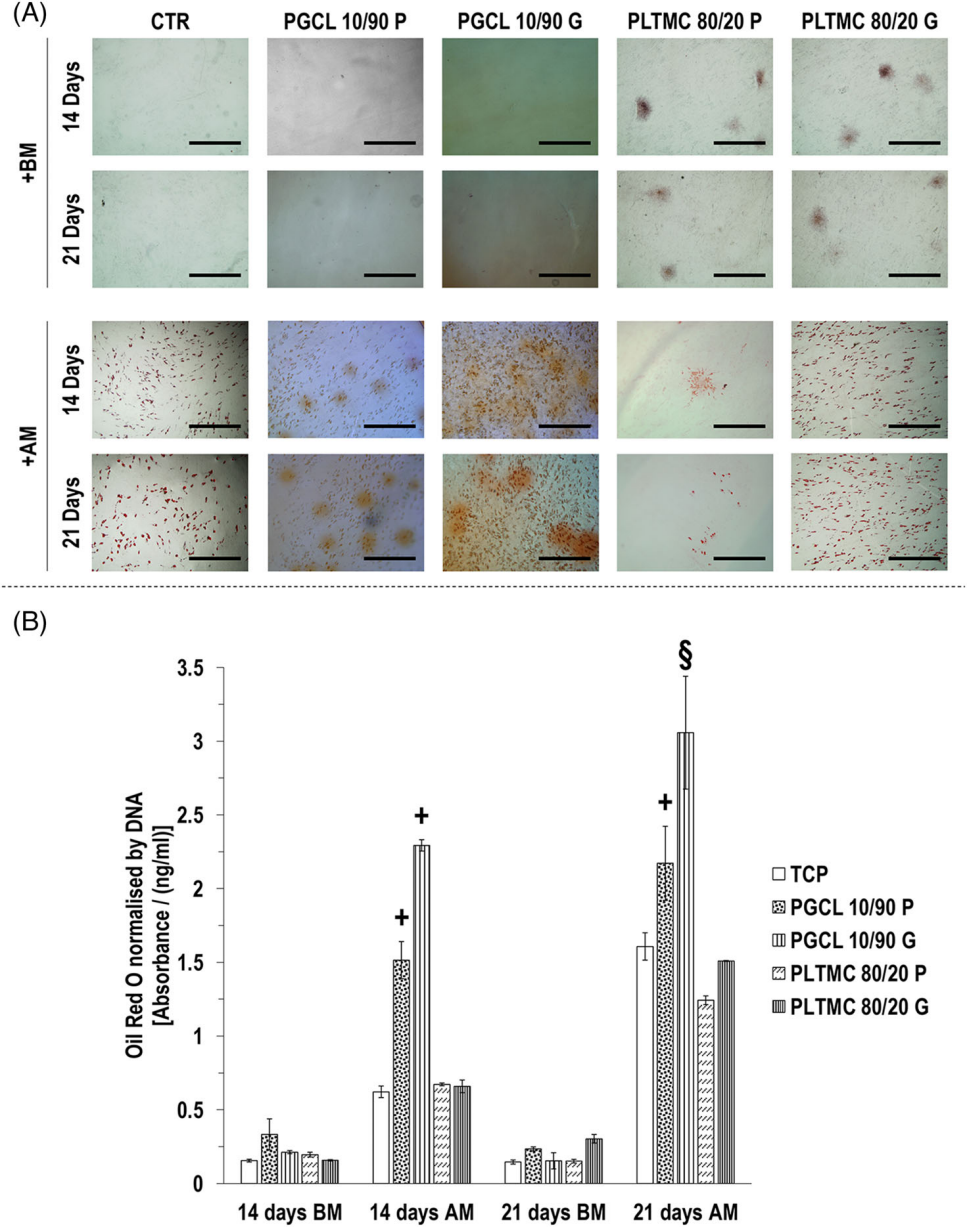
In adipogenic media on day 14 and day 21 Oil Red O staining (A) and complementary quantification (B) revealed that the PGCL 10/90 G, followed by the PGCL 10/90 P, induced the highest (*p <* 0.05) Oil Red O deposition. § indicates the highest (*p <* 0.05) value at a given timepoint. + indicates significantly (*p <* 0.05) higher value than the other groups at a given timepoint. # indicates the lowest (*p <* 0.05) value at a given timepoint. *N* = 4.

Oil Red O staining (Figure 5A) and complementary quantification (Figure 5B) revealed that in BM, on day 14 and day 21, all groups induced similar (*p >* 0.05) lipid deposition. Oil Red O staining (Figure 5A) and complementary quantification (Figure 5B) revealed that in adipogenic media, on day 14 and day 21, the PGCL 10/90 G [highest (*p* < 0.05) among all groups], followed by the PGCL 10/90 P, induced the highest (*p <* 0.05) lipid deposition and on day 21, the PLTMC 80/20 G induced significantly (*p <* 0.05) higher lipid deposition compared to PLTMC 80/20 P.

## 4 DISCUSSION

The combined effect of surface topography and substrate stiffness on stem cell function using biodegradable polymers is still under‐investigated, despite the fact that such polymers constitute the building blocks of most implantable regenerative medicine devices. Thus, herein, two biodegradable (PGCL 10/90 and PLTMC 80/20) polymeric substrates with 7 kPa and 12 kPa elastic modulus, respectively, and without and with anisotropic topography (groove width of 1.08 ± 0.09 μm and 1.01 ± 0.03 μm; groove depth of 1.46 ± 0.12 μm and 1.43 ± 0.10 μm; distance between groves of 1.64 ± 0.08 μm and 1.78 ± 0.05 μm, respectively) were fabricated and their effect in human bone marrow stem cell cultures was assessed.

### 4.1 Material characterization analysis

Starting with the characterization of the materials, microscopy analysis revealed that NIL allowed for the production of substrates with consistent surface topography and pattern fidelity. Indeed, NIL has become the method of choice for the scalable and reproducible production of substrates with sub‐micron topographical features [50, 51, 52, 53]. Thermal properties analysis made apparent that the planar and the grooved PGCL 10/90 substrates exhibited lower *T*_g_, Δ*H*_cc_, and *T*_m_ and higher *X*_c_ than their PLTMC 80/20 counterpart substrates, which is attributed to the presence of high amount of ε‐caprolactone inducing higher plasticization effect [54]. For both polymers, imprinting increased *T*_m_, suggesting that the crystalline structures have become more stable, both thermally and chemically due to the second heating of the polymeric substrates [55]. For both polymers, imprinting increased contact angle and decreased surface energy. These results are aligned with a previous report, where 2 μm gratings showed significantly higher contact angle compared to flat poly(vinyl alcohol) hydrogel samples [56]. The planar PLTMC 80/20 substrates exhibited higher contact angles and lower surface energy than the planar PGCL 10/90 substrates, which can be attributed to the lack of additional asymmetrical methyl groups in poly(glycolide) monomer making PGCL 10/90 more hydrophilic compared to very hydrophobic poly(lactide) and poly(trimethylene carbonate) monomers [57].

### 4.2 Cell morphometry analysis

Cytoskeleton clearly aligned parallel to the direction of the grooves as early as day 3, whilst nuclei did not elongate even after 7 days in culture. The influence of grooves/ridges dimensions on cell morphology and orientation has been extensively reported and have highlighted a strong influence of grooves width and depth on contact guidance [58, 59]. Although, the mechano‐transduction theory proposes that intracellular tension in elongated and aligned cytoskeleton actin filaments is transferred to the nucleus through cytoskeletal elements [28, 60, 61], this indifference in nuclei morphology may be attributed to the reduced compliance of nuclei to physical cues [62] and is in agreement with previous publications [28, 63].

### 4.3 Basic cellular function analysis

No significant differences in cell proliferation and metabolic activity were observed between the groups. Similarly, to our results, proliferation of hBMSCs was not affected by the use of grooved substrates [64], while other studies have shown enhanced hBMSCs proliferation by grooved topography [65, 66]. This inconsistency suggests that there are various factors at play in addition to the topography and rigidity, such as cell culture conditions and material's chemistry and degradation. The materials used herein are considered to have low degradation rate (the PGCL 10/90 and PLTMC 80/20 were resistant to degradation up to 21 days, showing no change in their thermal and mechanical properties [43]), way longer than then longest time point assessed herein (7 days), which explains their similar cytocompatibility for the duration of the experiments.

### 4.4 Stem cell differentiation analysis

After 21 days (longest time point assessed) in osteogenic culture, the TCP (elastic modulus ∼3 GPa) and the PLTMC 80/20 G (12 kPa) induced significantly higher calcium deposition and ALP activity than the other groups. This is in accordance with previous literature in the field showing that high in stiffness substrates [67, 68, 69, 70] and/or surfaces with topographical features [24, 26, 71–73] enhance in vitro osteogenesis. Interestingly, the PLTMC 80/20 G induced an improved (higher, but not significant) increase in calcium deposition and a significant increase in ALP activity over the planar PLTMC 80/20 calcium deposition, whilst the PGCL 10/90 G induced significantly lower calcium deposition and improved (higher, but not significant) ALP activity over the planar PGCL 10/90. Collectively these observations are suggestive of that there is a minimum effective substrate rigidity (∼12 kPa) to induce osteogenic differentiation, which can be further enhanced with surface topography. This is in agreement with previous publications, where substrates with 46.7 kPa rigidity and anisotropic topography (10 mm groove ridge width, 10 mm ditch width, and depth of 5 mm) were shown to enhance osteogenesis, whilst substrates with 6.1 kPa rigidity and no topography (planar PA hydrogel) did not [74].

After 21 days (longest time point assessed) of chondrogenic induction, the TCP (elastic modulus ∼3 GPa) and the PLTMC 80/20 G (12 kPa) induced significantly higher GAG deposition and the planar PGCL 10/90 showed the lowest GAG deposition than the other groups. Similarly, to osteogenic induction data, it has been well‐documented in the literature that stiff substrates are more chondrogenic than soft substrates [75]. It is interestingly to note that both grooved PLTMC 80/20 and PGCL 10/90 induced significantly higher GAG deposition than their planar counterparts, which also agrees with previously published reports showing enhanced chondrogenesis on grooved substrates [76] and bidirectionally aligned electrospun scaffolds [77]. Again, in this set of experiments, a complementary effect of surface topography to substrate rigidity is evidenced, as it has been argued before [42].

After 14 days (longest time point assessed) in BM, the rigid TCP downregulated 7(EGR1, EGR2, SCX, COL1A1, COL3A1, MKX, TNMD) genes and only upregulated 1 (TNC) gene, whilst in the tenogenic media, even the rigid TCP upregulated 4(SCX, COL1A1, MKX, TNMD) and downregulated 4(EGR1, EGR2, COL3A1, TNC) genes. These data indicate that the tenogenic media used is indeed suitable to induce tenogenic induction, considering that SCX, COL1A1, MKX, and TNMD are customarily used either assess tenogenic phenotype [78] or tenogenic induction [79]. At the same time point in the BM, the planar PGCL 10/90 upregulated 3(EGR1, COL3A1, TNC) genes, downregulated 4(EGR2, SCX, COL1A1, TNMD) genes and did not change 1 gene (MKX), whilst the grooved PLTMC 80/20 upregulated 3(EGR1, MKX, TNC) genes, downregulated 4(EGR2, SCX, COL1A1, COL3A1) genes and did not change 1(TNMD) gene. Similarly, in the tenogenic media, the planar PGCL 10/90 upregulated 7(EGR1, EGR2, COL1A1, COL3A1, MKX, TNC, TNMD) genes and downregulated 1 (SCX) gene, whilst the grooved PLTMC 80/20 upregulated 7(EGR1, EGR2, SCX, COL1A1, COL3A1, MKX, TNMD) genes and downregulated 1(TNC) gene. All these observations suggest that low rigidity without topography (in the case of planar 7 kPa PGCL 10/90) may be the driving factor in tenogenic induction and when rigidity is increased, surface topography can be used to counterbalance it (in the case of the grooved 12 kPa PLTMC 80/20). This is in agreement with a previous publication, where topographical features although increased the expression of tenogenic genes, the high in rigidity poly(lactic‐*co*‐glycolic acid) 85/15 substrates used also resulted in increased expression of osteogenic and chondrogenic genes in tenocyte cultures [28]. It is also important to note that in another paper, tenocytes cultured on 50 kPa anisotropic substrates maintained most closely their native phenotype, in comparison to cells grown on stiffer without/with topography substrates [63].

After 21 days (longest time point assessed) in adipogenic media, both the planar and the grooved PGCL 10/90 substrates, being the softest (7 kPa), induced significantly higher oil droplet deposition than the other substrates and within them, the grooved PGCL 10/90 induced significantly higher droplet deposition than the planar. Both these observations are in agreement with previous data in the field. Indeed, numerous publications have shown soft substrates to be more adipogenic than stiff substrates [27, 80, 81] and topographical features to enhance adipogenic potential [26, 72, 82, 83].

## 5 CONCLUSIONS

The combined effect of surface topography and substrate stiffness on stem cell function using biodegradable polymers is still under‐investigated. To this end, we assessed the influence of anisotropic and planar surface topography of 7 kPa PGCL 10/90 (groove width of 1.08 ± 0.09 μm; groove depth of 1.46 ± 0.12 μm; distance between groves of 1.64 ± 0.08 μm) and 12 kPa PLTMC 80/20(groove width of 1.01 ± 0.03 μm; groove depth of 1.43 ± 0.10 μm; distance between groves of 1.78 ± 0.05 μm) on human bone marrow stem cell response. Our data indicate that anisotropic surface topography is key modulator of cell morphology. High substrate rigidity combined with surface topography appear to enhance osteogenic and chondrogenic differentiation. Low substrate rigidity alone or in combination with surface topography appear to favor tenogenesis and adipogenesis. Collectively our data further advocate the use of biomaterials with tissue‐specific physical properties.

## AUTHOR CONTRIBUTIONS

Sofia Ribeiro and Dimitrios I. Zeugolis designed the study and wrote the manuscript. Sofia Ribeiro carried out experiments and analyzed data. Emanuel M. Fernandes was involved in the processing of the polymeric substrates. Eugenia Pugliese optimized tenogenic differentiation method. Stefanie H. Korntner optimized qPCR experiments. Alan O'Riordan provided the silicon master stamps with grooved topography. All authors discussed the data and approved the final version of the manuscript.

## CONFLICTS OF INTEREST

S.R. was an early career researcher recruited at Sofradim Production, Medtronic, France and registered for PhD at NUI Galway, Ireland. Y.B. is an employee of Sofradim Production, Medtronic, France. E.P., S.H.K., E.M.F., M.E.G., R.L.R., A.O'R, and D.I.Z. declare no conflicts of interest.

## Supporting information

SUPPORTING INFORMATION

## Data Availability

The raw and processed data required to reproduce these findings are available on request from S.R.
