# Supplementary material for: Assessing the combined effect of surface topography and substrate rigidity in human bone marrow stem cell cultures
Source: Eng Life Sci. 2022 Sep 13;22(10):619–33. doi: 10.1002/elsc.202200029 (PMC9550738; doi:10.1002/elsc.202200029)

# Supplementary Information

**Assessing the combined effect of surface topography and substrate rigidity in human bone marrow stem cell cultures**

Sofia Ribeiro ^1, 2^, Eugenia Pugliese ^2^, Stefanie H. Korntner ^2^, Emanuel M. Fernandes ^3, 4^, Manuela E. Gomes ^3, 4^, Rui L. Reis ^3, 4^, Alan O'Riordan ^5^, Yves Bayon ^1^, Dimitrios I. Zeugolis ^2, 6^

^1^ Medtronic, Sofradim Production, Trevoux, France

^2^ Regenerative, Modular & Developmental Engineering Laboratory (REMODEL) and Science Foundation Ireland (SFI) Centre for Research in Medical Devices (CÚRAM), National University of Ireland Galway (NUI Galway), Galway, Ireland

^3^ 3B's Research Group, I3Bs – Research Institute on Biomaterials, Biodegradables and Biomimetics, University of Minho, Headquarters of the European Institute of Excellence on Tissue Engineering and Regenerative Medicine, AvePark, Parque de Ciência e Tecnologia, Zona Industrial da Gandra, 4805-017 Barco, Guimarães, Portugal

^4^ ICVS/3B’s – PT Government Associate Laboratory, Braga/Guimarães, Portugal

^5^ Tyndall National Institute, Cork, Ireland

^6^ Regenerative, Modular & Developmental Engineering Laboratory (REMODEL), Charles Institute of Dermatology, Conway Institute of Biomolecular & Biomedical Research and School of Mechanical & Materials Engineering, University College Dublin (UCD), Dublin, Ireland

**Correspondence:** Prof Dimitrios I. Zeugolis (dimitrios.zevgolis@ucd.ie). REMODEL, UCD, Belfield, D04 V1W8, Dublin 4, Ireland.

**Supplementary Table S1:** Primary antibodies used for FACS analysis.

| **Antibody** | **Clone** | **Host** | **Isotype** |
| --- | --- | --- | --- |
| CD31 | WM59 | Human | IgG1 |
| CD44 | G44-26 | Human | IgG2bk |
| CD45 | HI30 | Human | IgG1 |
| CD73 | AD2 | Human | IgG1 |
| CD90 | 5E10 | Human | IgG1 |
| CD105 | 266 | Human | IgG1 |
| CD146 | P1H12 | Human | IgG2a |

**Supplementary Table S2:** TaqMan primer probe assay IDs used for gene analysis.

| **Gene** | **AssayID** |
| --- | --- |
| HPRT1 | Hs.PT.39a.22214821 |
| TBP | Hs.PT.39a.22214825 |
| EIF2B1 | Hs.PT.58.28166370 |
| COL1A1 | Hs.PT.56a.40971026 |
| EGR1 | Hs.PT.58. 40805543.g |
| EGR2 | Hs.PT.58.40402328 |
| SCX | Hs03054634_g1 |
| COL3A1 | Hs.PT.58.4249241 |
| MKX | Hs.PT.58.40174404 |
| TNC | Hs.PT.58.2529606 |
| TNMD | Hs.PT.58.14567828 |

**Supplementary Figure S1:** Flow cytometry analysis of the hBMSCs (passage 3) revealed that most hBMSCs at passage 3 expressed CD73 (89.7 %), CD90 (99.9 %), CD105 (96.2 %) and CD44 (99.8 %) and did not express CD45 (0.095 %), CD31 (2.88 %) and C146 (6.31 %). The grey histogram corresponds to the isotype control and the blue histogram correspond to the specific marker. N = 1


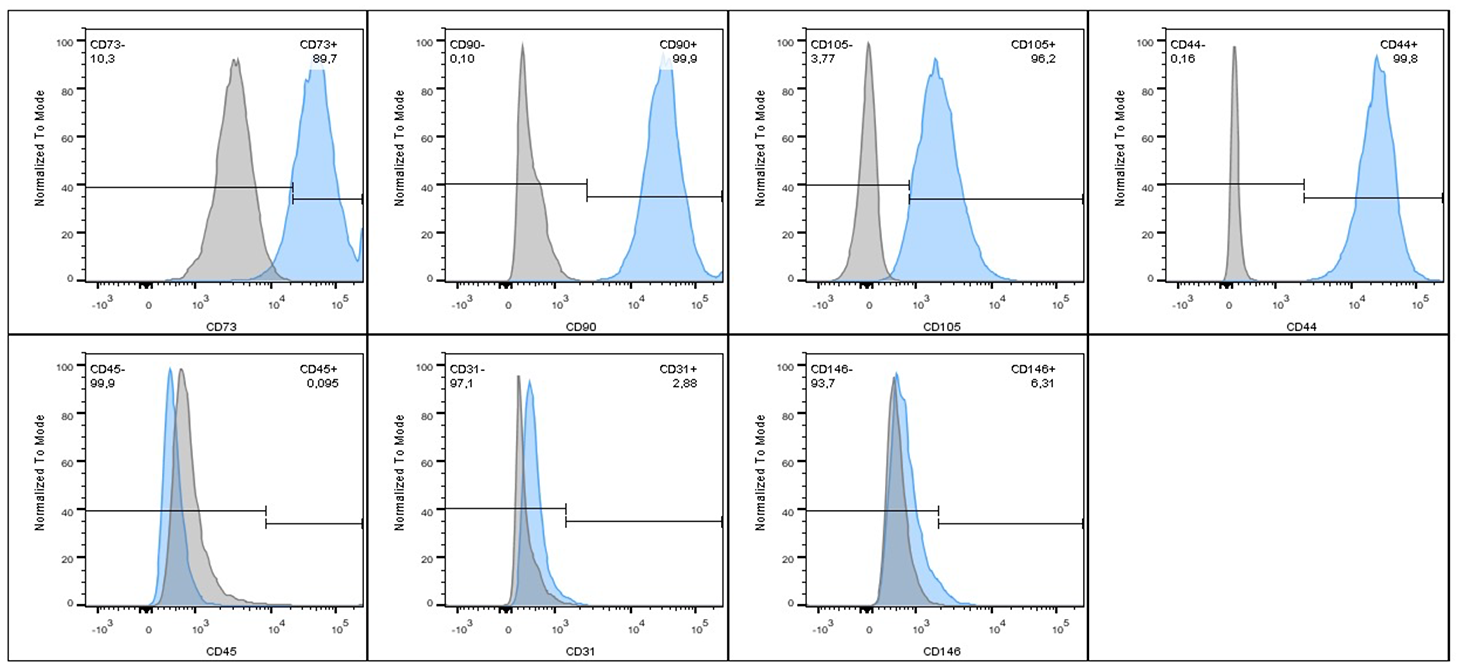


**Supplementary Figure S2:** Flow cytometry analysis of the hBMSCs-SF (passage 3) revealed that most hBMSCs-SF at passage 3 expressed CD73 (100 %), CD90 (100 %), CD105 (100 %), CD44 (100 %) and did not express CD45 (0.18 %), CD31 (0.31 %), but did express C146 (77.2 %) due to the absence of serum for one full passage during hBMSCs-SF isolation. The grey histogram corresponds to the isotype control and the blue histogram correspond to the specific marker. N = 1


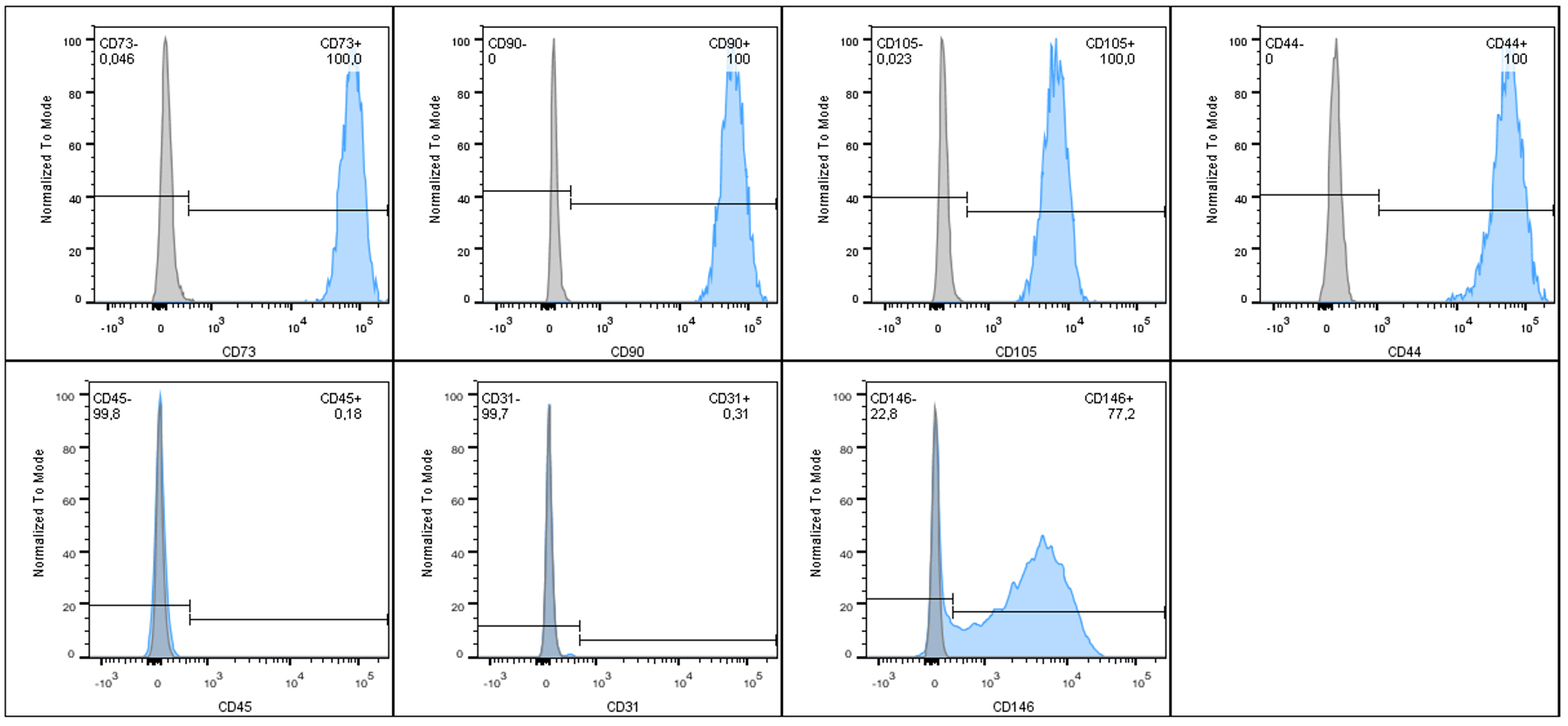


**Supplementary Figure S3:** Indicative DSC second heating curves of planar (full line) and grooved (dashed line) polymeric substrates PGCL 10/90 (A) and PLTMC 80/20 (B).


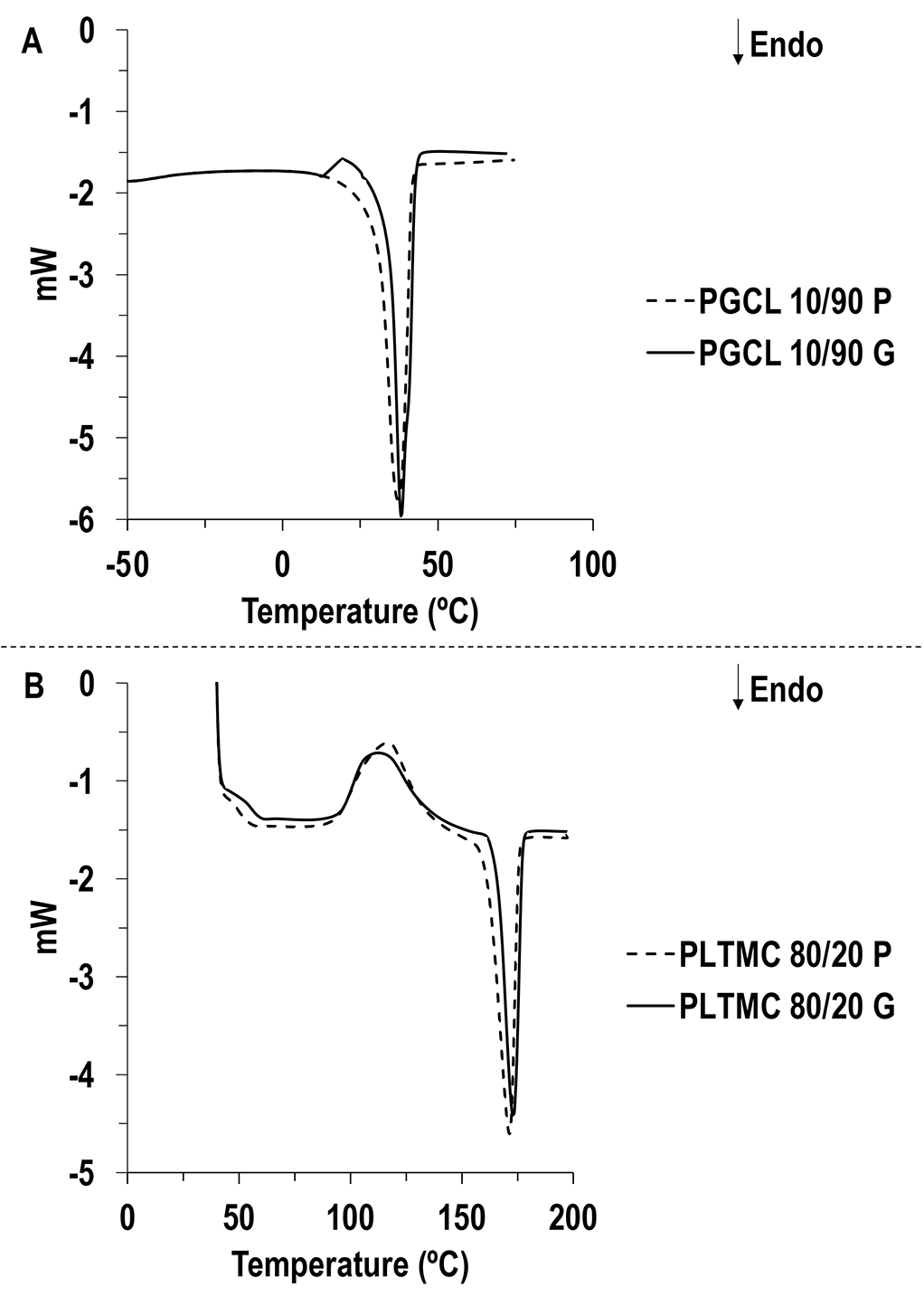


**Supplementary Figure S4:** Immunocytochemistry analysis revealed that the cells oriented randomly on all planar substrates and aligned parallel to the orientation of the grooves on all grooved substrates. Cytoskeleton: red. Nuclei: blue. White arrow indicates direction of surface topography. Scale bars: 100 µm.


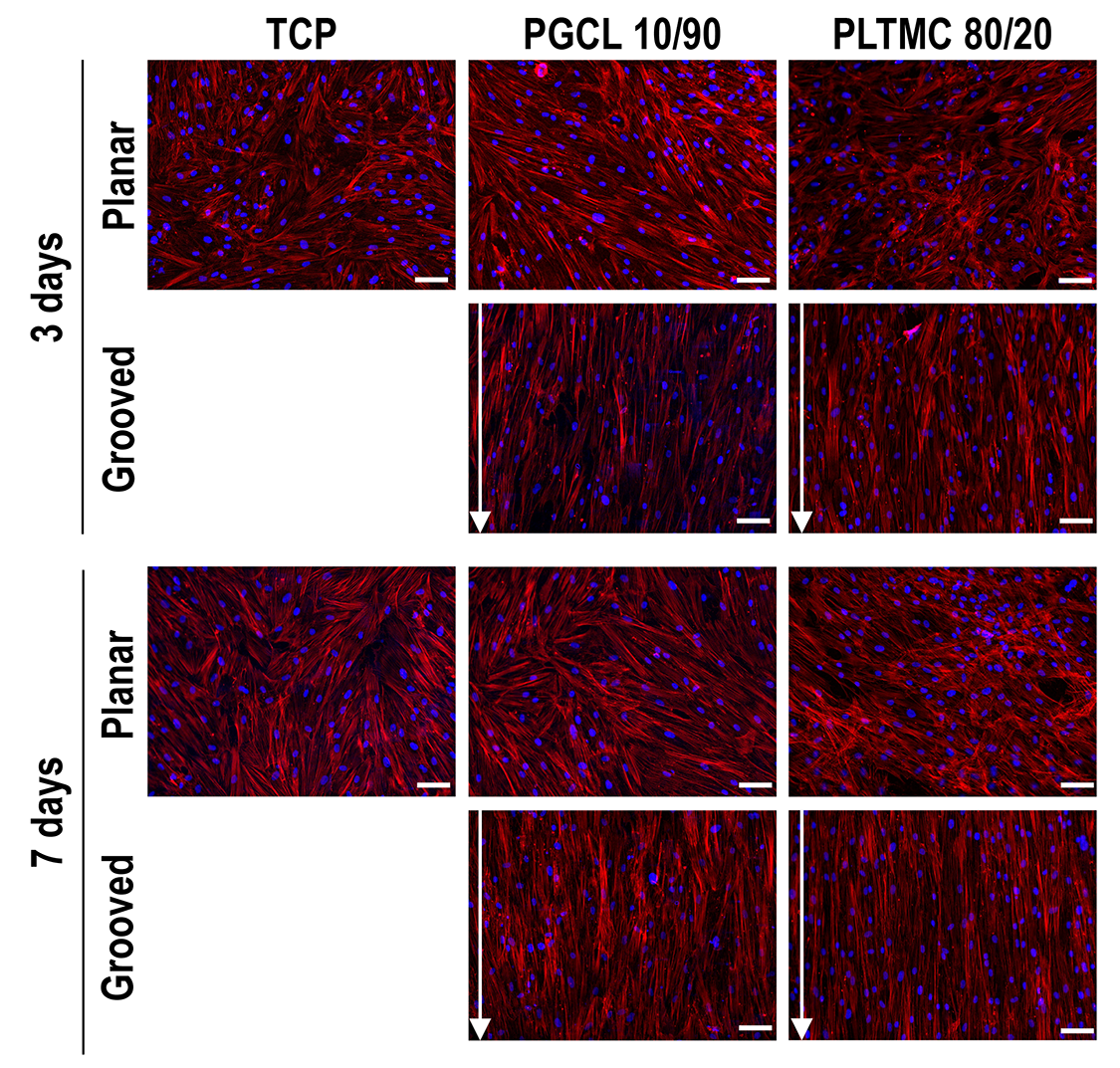


**Supplementary Figure S5:** hBMSCs nuclear orientation/alignment (A) and nuclear aspect ratio quantification (B) at day 3 and at day 7, revealed that cells adopted a random orientation on planar films and a bidirectional morphology, parallel to the orientation of the groves, on the grooved substrates at both timepoints.


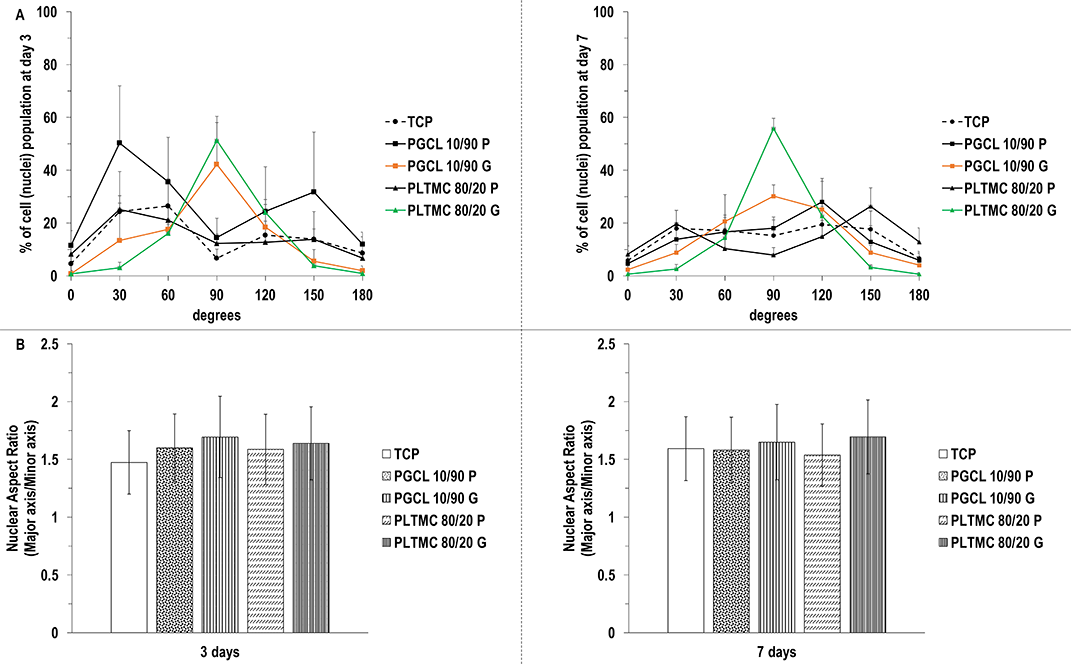


**Supplementary Figure S6:** Cell proliferation (A) and metabolic activity (B) analyses revealed no significant differences between the groups.


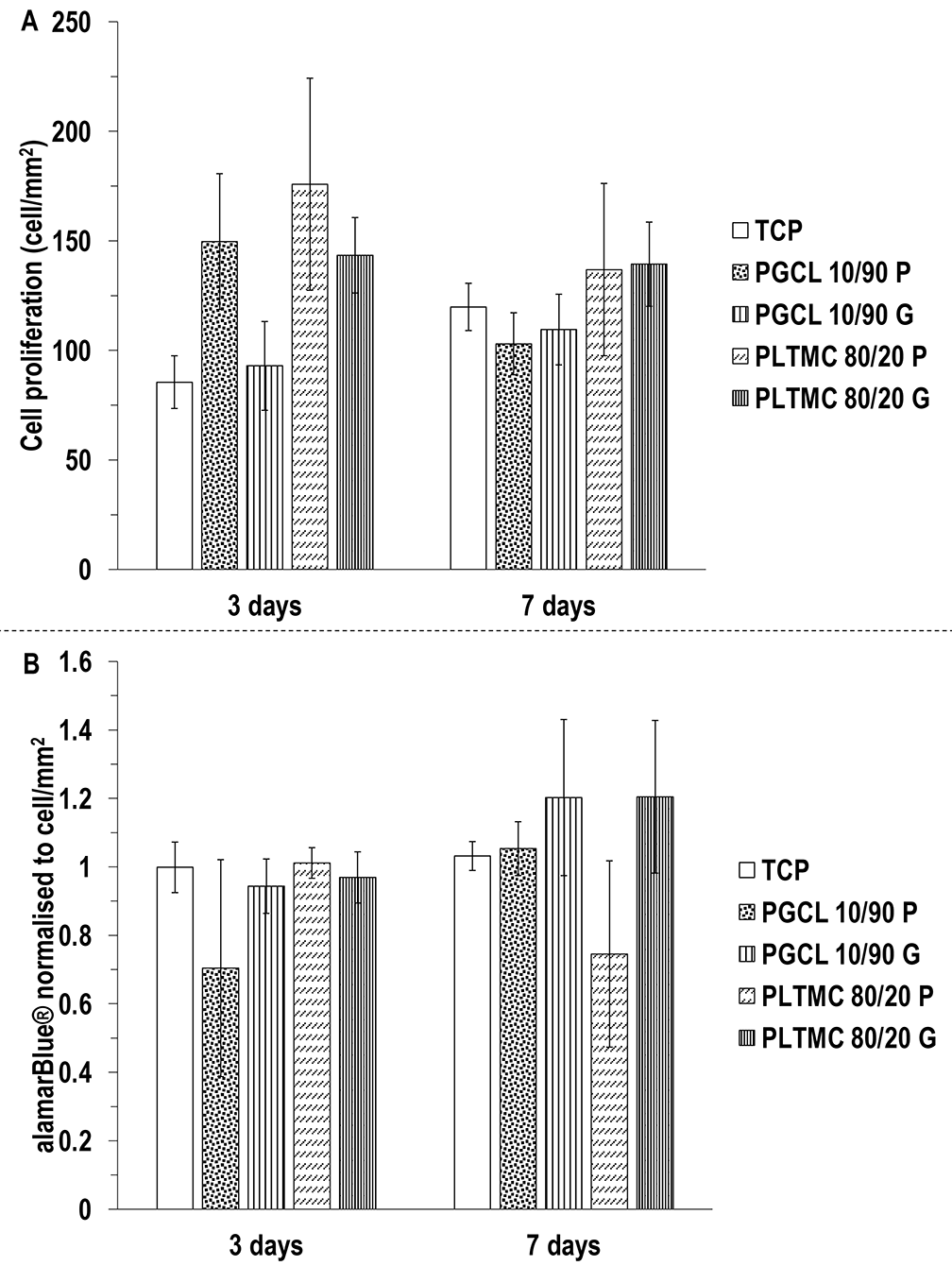

Supplement: Supplementary file 1 — SUPPORTING INFORMATION [file ELSC-22-619-s001.docx]
